# Lombosciatique révélant un épendymome intramédullaire

**DOI:** 10.11604/pamj.2017.26.171.11633

**Published:** 2017-03-24

**Authors:** Zeineb Alaya, Walid Osman

**Affiliations:** 1 Service de Rhumatologie, Hôpital Farhat Hached, Sousse, Tunisie; 2 Service d’Orthopédie, Hôpital Sahloul, Sousse, Tunisie

**Keywords:** Lombosciatique, IRM, tumeur rachidienne, épendymome, Lumbosciatica pain, MRI, spinal tumor, ependymoma

## Image en médecine

We here report the case of a 68-year old female patient presenting with inflammatory poorly systematized bilateral lumbosciatica, without vesico-sphincteric disorders or associated general symptoms evolving for 4 months. Physical exam showed gait with little steps with trunk anteflexion, disappearance of lumbar lordosis, lumbar spine stiffness, positive bilateral Lasègue's test and hyperactive osteotendinous reflexes in lower limbs without motor and sensory deficit or saddle block anesthesia. Laboratory tests did not show any inflammatory syndrome. Lumbar spine X-rays were normal. Given the abnormal clinical picture, spinal MRI was performed which showed oval intracanalar mass behind L4 vertebral body, measuring 3 cm height with discrete hypersignal on T1 (A) and with hypersignal on T2 (B), enhanced after gadolinium administration and containing superior polar cystic component. The patient underwent tumor resection in orthopaedics. Histological examination of the surgical specimen revealed myxopapillary ependymomas.

**Figure 1:**
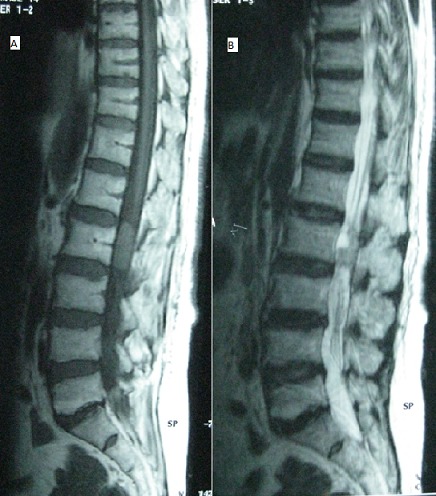
IRM rachidienne: présence en intra-canalaire d’une formation ovalaire en arrière du corps vertébral de L4, mesurant 3 cm de hauteur qui apparait en discret hypersignal T1 (A) et en hypersignal T2 (B), se réhaussant après injection de gadolinium et comportant une composante kystique polaire supérieure

